# Inhibition of Soluble Epoxide Hydrolase Attenuates High-Fat-Diet–Induced Hepatic Steatosis by Reduced Systemic Inflammatory Status in Mice

**DOI:** 10.1371/journal.pone.0039165

**Published:** 2012-06-14

**Authors:** Yan Liu, Huaixin Dang, Dan Li, Wei Pang, Bruce D. Hammock, Yi Zhu

**Affiliations:** 1 Department of Physiology and Pathophysiology, Key Laboratory of Molecular Cardiovascular Sciences of Education Ministry, Peking University Health Science Center, Beijing, China; 2 Department of Entomology and Cancer Center, University of California Davis, Davis, California, United States of America; The Chinese University of Hong Kong, Hong Kong

## Abstract

Non-alcoholic fatty liver disease is associated with obesity and considered an inflammatory disease. Soluble epoxide hydrolase (sEH) is a major enzyme hydrolyzing epoxyeicosatrienoic acids and attenuates their cardiovascular protective and anti-inflammatory effects. We examined whether sEH inhibition can protect against high-fat (HF)-diet–induced fatty liver in mice and the underlying mechanism. Compared with wild-type littermates, sEH-null mice showed lower diet-induced lipid accumulation in liver, as seen by Oil-red O staining and triglycerides levels. We studied the effect of sEH inhibition on diet-induced fatty liver by feeding C57BL/6 mice an HF diet for 8 weeks (short-term) or 16 weeks (long-term) and administering t-AUCB, a selective sEH inhibitor. sEH inhibition had no effect on the HF-diet–increased body and adipose tissue weight or impaired glucose tolerance but alleviated the diet-induced hepatic steatosis. Adenovirus-mediated overexpression of sEH in liver increased the level of triglycerides in liver and the hepatic inflammatory response. Surprisingly, the induced expression of sEH in liver occurred only with the long-term but not short-term HF diet, which suggests a secondary effect of HF diet on regulating sEH expression. Furthermore, sEH inhibition attenuated the HF-diet–induced increase in plasma levels of proinflammatory cytokines and their mRNA upregulation in adipose tissue, which was accompanied by increased macrophage infiltration. Therefore, sEH inhibition could alleviate HF-diet–induced hepatic steatosis, which might involve its anti-inflammatory effect in adipose tissue and direct inhibition in liver. sEH may be a therapeutic target for HF-diet–induced hepatic steatosis in inhibiting systemic inflammation.

## Introduction

Obesity, a chronic inflammatory condition, is becoming a major health issue worldwide and is closely associated with metabolic disorders such as diabetes, coronary heart disease and fatty liver disease [1]. Non-alcoholic fatty liver disease (NAFLD) is one of the most common forms of chronic liver disease and ranges from pure fatty liver to the more severe nonalcoholic steatohepatitis and cirrhosis, with build-up in liver cells of excess neutral lipids, mainly triglycerides, not due to alcohol consumption. NAFLD is also considered a risk factor for diabetes and cardiovascular diseases, independent of other traditional risk factors [2]. With the “two-hit” hypothesis of the progression of NAFLD, insulin resistance and the consequent triglycerides accumulation are considered the first hit and oxidative stress, endoplasmic reticulum stress, increased proinflammatory cytokines expression and cellular injury the second hit [3].

Obesity and NAFLD are strongly linked [4]. Increased delivery of non-esterified fatty acids from adipose tissue in obese individuals is an important source of excessive lipid deposition in hepatocytes. Approximately 60% of fat accumulating in the liver is from adipose tissue [5]. As well, in animal models of high fat (HF)-diet–induced obesity and metabolic disorder, increased fat in the diet is another critical source of excess fat in the liver [5]. Moreover, adipose tissue is considered an endocrine organ that secretes proinflammatory cytokines such as tumor necrosis factor α (TNF-α) and interleukin 6 (IL-6), thus contributing to the first and second hits of NAFLD [6], [7], [8]. Therefore, treatment strategies specific to NAFLD include improving insulin sensitivity and inflammatory status, as well as modifying underlying metabolic risk factors.

Recently, soluble epoxide hydrolase (sEH, *EPHX2*), functioning to enzymatically hydrolyze epoxyeicosatrienoic acids (EETs) and other fatty acid epoxides, has attracted great interest as a potential therapeutic target for renal, cardiovascular and inflammatory diseases [9]. sEH is distributed in various tissues and highly expressed in liver, kidney and the cardiovascular system. It is mainly localized in the cytosol of the cell, which contains the EH catalytic domain in the C-terminal region, and hydrolyzes EETs and other fatty acid epoxides to their respective diols [10], [11]. EETs have significant protective effects in regulating vascular, cardiac and renal physiologic functions [12]. Moreover, EETs are considered anti-inflammatory agents by their blocking the activation of NF-κB [13]. Inflammation is believed an initial factor of hepatic lipid accumulation. sEH inhibition elevates the level of EETs, and sEH activity is considered a major determinant of EET bioavailability [9], [14]. The substrates of sEH include the epoxy products of omega-3 polyunsaturated fatty acids, especially docosahexaenoic acid (DHA) and eicosapentaenoic acid (EPA) [15], which play a critical role in relieving hepatic steatosis by regulating nuclear factors involved in fatty acid de novo synthesis and utilization [16]. The epoxy metabolites of DHA/EPA have some beneficial effects, such as anti-inflammatory effects [15], [17].

Although sEH inhibition can improve cardiovascular and renal diseases, the therapeutic potential of sEH inhibition in diet-induced obesity and lipid metabolism disorder is still largely unknown [9]. Investigation of genetic polymorphisms in sEH has suggested its association with plasma triglycerides homeostasis [18]. sEH expression was found lower in adipose tissue than in liver and kidney, but total adipose sEH activity was selectively increased during the development of obesity in mice fed an HF diet [19]. In a food-induced metabolic-syndrome rat model, chronic oral treatment with *trans*-4-[4-(3-adamantan-1-ylureido)-cyclohexyloxy]-benzoic acid (t-AUCB), a potent sEH inhibitor [20], alleviated the signs of metabolic syndrome, including lipid abnormalities and structural and functional changes in the liver [21].

Considering the relevance of sEH for inflammation, cardiovascular disorders, potential lipid abnormalities, and regulation in adipose tissue, we investigated the role of sEH in HF-diet–induced lipid abnormalities and the associated changes in liver by treatment with sEH inhibition, gene knockout or overexpression with recombinant human sEH in mice. sEH inhibition could attenuate HF-diet–induced inflammation in both adipose tissue and liver, which may be a therapeutic strategy for treating fatty liver.

## Results

### sEH Deficiency or Activity Inhibition Reduced HF-diet–induced Level of Hepatic Triglycerides

**Figure 1:**
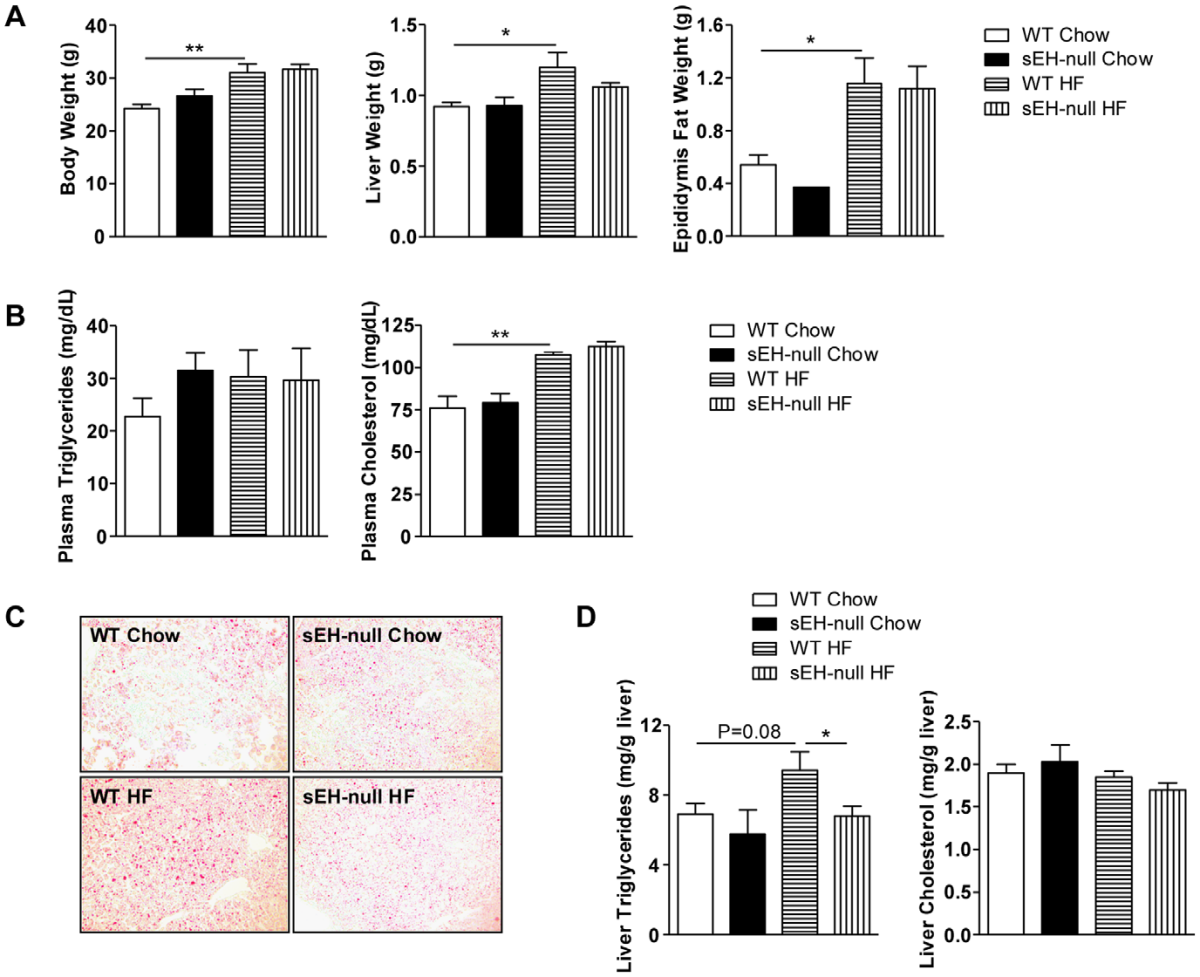
sEH deficiency ameliorated high-fat (HF)-diet–induced hepatic steatosis in mice. Wild type (WT) littermates and sEH-null mice were fed a regular chow or HF diet for 8 weeks. (WT Chow, WT HF: n=6; sEH-null Chow, sEH-null HF: n=8) (A) Body weight, liver weight and epididymal fat weight. (B) Plasma levels of triglycerides and cholesterol. (C) Oil-red O staining in liver sections. (D) Levels of cholesterol and triglycerides in liver. Data are mean ± SEM (* P<0.05, ** P<0.01).

*EPHX2* gene polymorphism is associated with plasma lipid and lipoprotein level [18], which suggests that sEH may play a role in lipid metabolism. We studied the role of sEH in lipid metabolism and the underlying mechanism in HF-diet–induced lipid metabolism disorder in mice with whole-body knockout of *Ephx2* (sEH null) [22] and their wild-type (WT) littermates. HF diet for 8 weeks increased the body weight and weight of liver and fat tissue in WT and sEH-null mice (Fig. 1A). Plasma levels of triglycerides and cholesterol were not affected by an HF diet in sEH-null mice (Fig. 1B). However, lipid accumulation in liver was lower in sEH-null than WT mice with an HF diet (Fig. 1C), and triglycerides content was lower in sEH-null liver (Fig. 1D).

**Figure 2:**
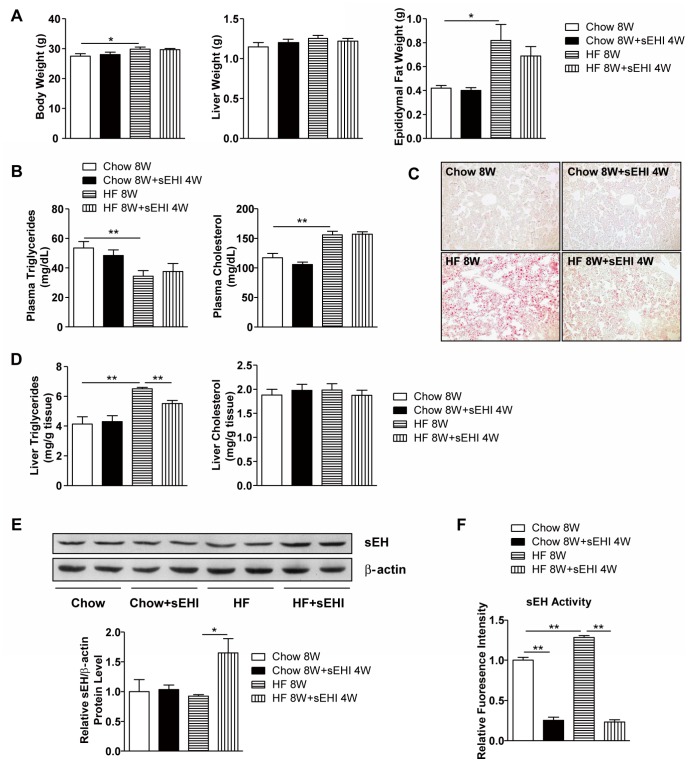
4-week sEH inhibitor administration attenuated 8-week HF-diet–induced triglycerides accumulation in mouse liver. Male C57BL/6 mice were fed regular chow or a HF diet for 8 weeks with or without sEH inhibitor (sEHI) t-AUCB administration in drinking water from week 5 (n=8 mice/group). (A) Body weight, liver weight and epididymal fat weight. (B) Plasma levels of triglycerides and cholesterol. (C) Oil-red O staining in liver sections. (D) Levels of triglycerides and cholesterol in liver. (E) Western blot analysis of protein levels of sEH and β-actin as a normalization control in liver. (F) sEH activity in liver. Data are mean ± SEM. (* P<0.05, ** P<0.01).

To study whether sEH inhibition can reverse the effect of an HF diet on fatty liver, we fed mice an HF diet for 8 weeks and administered a selective sEH inhibitor, t-AUCB, in drinking water to half of the mice for 4 weeks starting from week 5. t-AUCB had no effect on HF-diet–increased body weight and fat tissue weight or plasma level of cholesterol and triglycerides (Fig. 2A, B) but reduced the HF-diet–induced mild hepatic steatosis (Fig. 2C, D). Of note, neither sEH deficiency nor activity inhibition altered the impaired glucose tolerance and insulin resistance in mice (Fig. S1). To determine whether an HF diet regulated sEH expression in the liver, which may play a role in lipid metabolism, we measured the protein expression of sEH in the liver and found no effect of the diet on sEH expression and slightly increased sEH activity (Fig. 2E, F). Hydrolase activity of sEH in liver was largely inhibited by sEH inhibition, but protein levels of sEH were increased, perhaps through a feedback mechanism. Thus, sEH expression does not play a major role in HF-diet–induced hepatic steatosis in these mice.

### sEH Inhibition Blocked HF-diet–induced Inflammation

**Figure 3:**
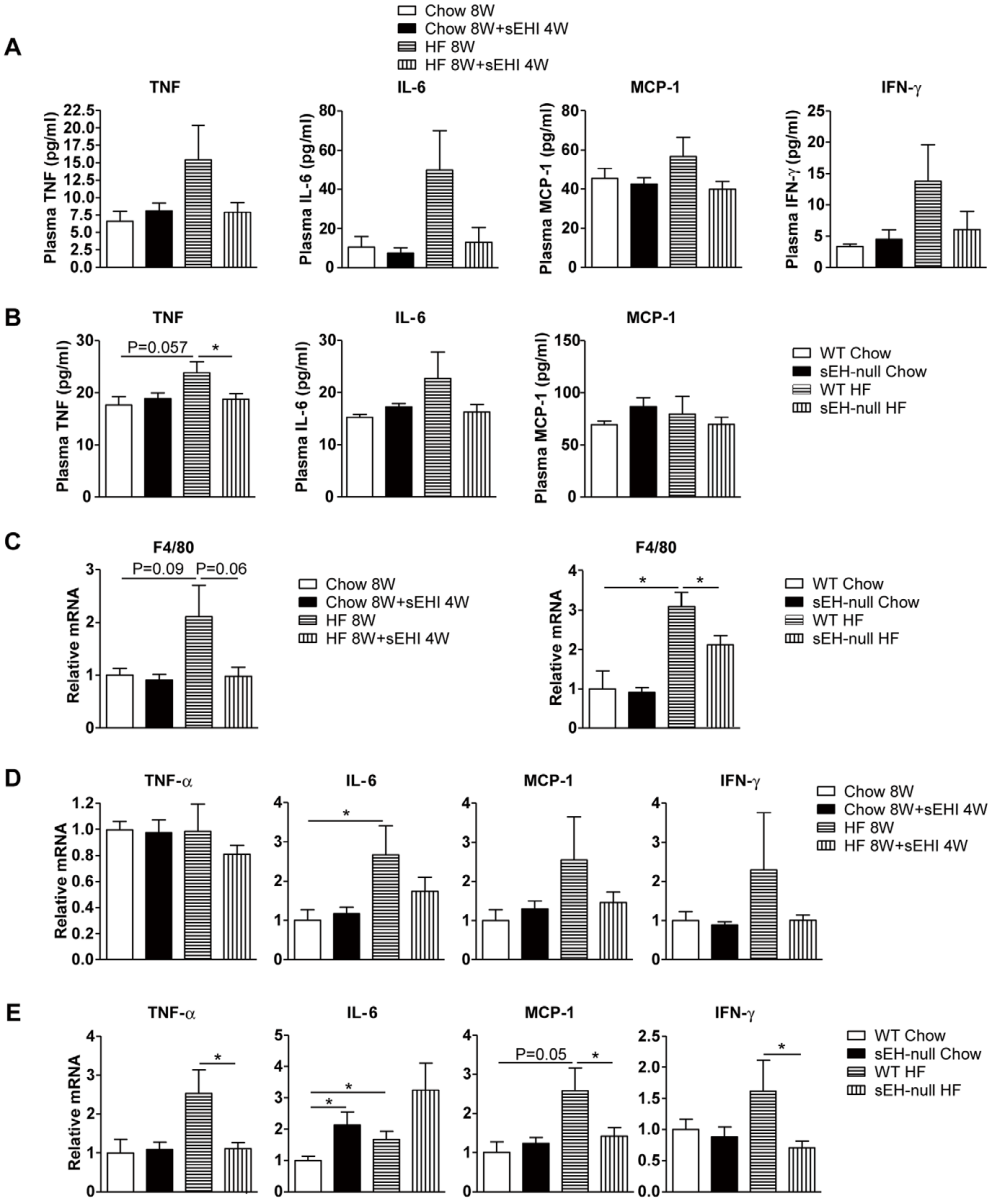
sEH inhibition or deficiency could block 8-week HF-diet–induced inflammation. (A) Proinflammatory cytokine levels in plasma of mice fed an 8-week chow or HF diet with or without 4-week t-AUCB. (B) Proinflammatory cytokine levels in plasma of WT and sEH-null mice fed an 8-week regular chow or HF diet. (C) mRNA levels of macrophage marker F4/80 in epididymal fat tissue. mRNA levels of tumor necrosis factor α (TNF-α) and interleukin 6 (IL-6), interferon γ (IFN-γ), monocyte chemoattractant protein 1 (MCP-1) in epididymal fat tissue of (D) mice fed an 8-week chow or HF diet with or without 4-week sEH inhibitor and (E) WT or sEH-null mice fed a chow or HF diet. Data are mean ± SEM. (* P<0.05, ** P<0.01).

To further investigate the mechanism of sEH inhibition in regulating hepatic lipid metabolism, we detected the expression of genes involved in fatty acid synthesis, including liver X receptor (LXR), carbohydrate response element binding protein (ChREBP), sterol regulatory element binding protein 1 (SREBP1), fatty acid synthase (FAS) and acetyl-Coenzyme A carboxylase 1 (ACC1), as well as genes involved in fatty acid β-oxidation, including peroxisome proliferator-activated receptor α (PPARα), carnitine palmitoyltransferase 1A (CPT1A), and acyl-CoA oxidase 1 (ACO1). The mRNA levels of these genes were not affected by sEH inhibition with an HF diet (Fig. S2A, B). Given that chronic inflammation in diet-induced obesity is associated with lipid metabolism, we next measured the plasma levels of proinflammatory cytokines, including TNF-α, IL-6, monocyte chemoattractant protein 1 (MCP-1) and interferon γ (IFN-γ). The levels of these cytokines were elevated but not significantly in WT mice fed an HF diet and were attenuated by sEH inhibition (Fig. 3A); similar patterns were found in sEH-null mice (Fig. 3B). Levels of TNF-α, MCP1 and IFN-γ were significantly lower in sEH-null than WT mice fed an HF diet (Fig. 3B).

Because mRNA levels of TNF-α and IL-6 in the liver were not altered by an HF diet or sEH inhibition (Fig. S2C), liver might be the targeted organ, instead of the source, of the increased levels of circulating inflammatory cytokines. Infiltrated inflammatory cells in adipose tissue and adipocytes were reported to be the major source of circulating inflammatory cytokines such as IL-6 [23], which suggests that adipose tissue is an important target for HF-diet–induced inflammation. Therefore, we detected the mRNA levels of the macrophage marker F4/80 in epididymal fat tissue of mice and found increased levels in HF-diet groups and reduced levels in both sEH-null and sEHI groups (Fig. 3C). Furthermore, 4-week sEH inhibition decreased, although not significantly, the expression of TNF-α, IL-6, IFN-γ and MCP-1 upregulated by the HF diet in adipose tissue (Fig. 3D). Similar results were observed in sEH-null mice (Fig. 3E). Therefore, the effect of sEH inhibition or sEH deficiency on HF-diet–induced lipid accumulation in the liver might result from anti-inflammatory effects in peripheral tissues, especially white adipose tissue, which is more sensitive to an HF diet than liver.

### Long-term sEH Inhibition Attenuated HF-induced Hepatic Steatosis

**Figure 4:**
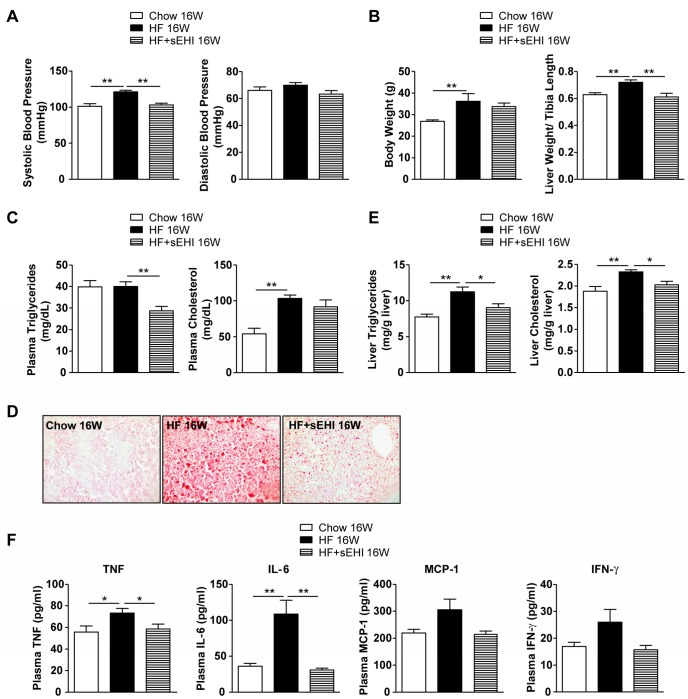
Full-term sEH inhibitor administration attenuated 16-week HF-diet–induced hepatic steatosis. Male C57BL/6 mice were fed a regular chow or HF diet for 16 weeks with or without sEHI t-AUCB in drinking water starting from 3 days before diet (Chow 16W: n=10, HF 16W: n=15, HF 16W+sEHI 16W: n=11). (A) Systolic and diastolic blood pressure. (B) Body weight and ratio of liver weight compared to tibia length. (C) Plasma levels of triglycerides and cholesterol. (D) Oil-red O staining in liver sections. (E) Levels of triglycerides and cholesterol in liver. (F) Proinflammatory cytokine levels in plasma. Data are mean ± SEM. (* P<0.05, ** P<0.01).

To explore the long-term protective effect of sEH inhibition on inflammation and hepatic lipid metabolism, we fed 8-week old male C57BL/6 mice an HF diet for 16 weeks, and half of the mice received the sEH inhibitor t-AUCB in drinking water beginning at 3 days before the diet. Long-term HF diet elevated systolic blood pressure (Fig. 4A), body weight and liver weight (Fig. 4B), plasma levels of cholesterol (Fig. 4C), and hepatic steatosis (Fig. 4D), impaired glucose tolerance and induced hyperinsulinemia (Fig. S3). Although sEH inhibition did not change HF-diet–increased plasma cholesterol level and insulin resistance, the elevated blood pressure, hepatic steatosis, and liver content of triglycerides and cholesterol were significantly attenuated with sEHI treatment (Fig. 4E). Moreover, the elevated plasma levels of triglycerides and proinflammatory cytokines such as TNF-α and IL-6 were attenuated by sEH inhibition (Fig. 4C, F).

### sEH Inhibition Decreased Long-term HF-diet–induced Activation of Inflammatory Pathways in Liver

**Figure 5:**
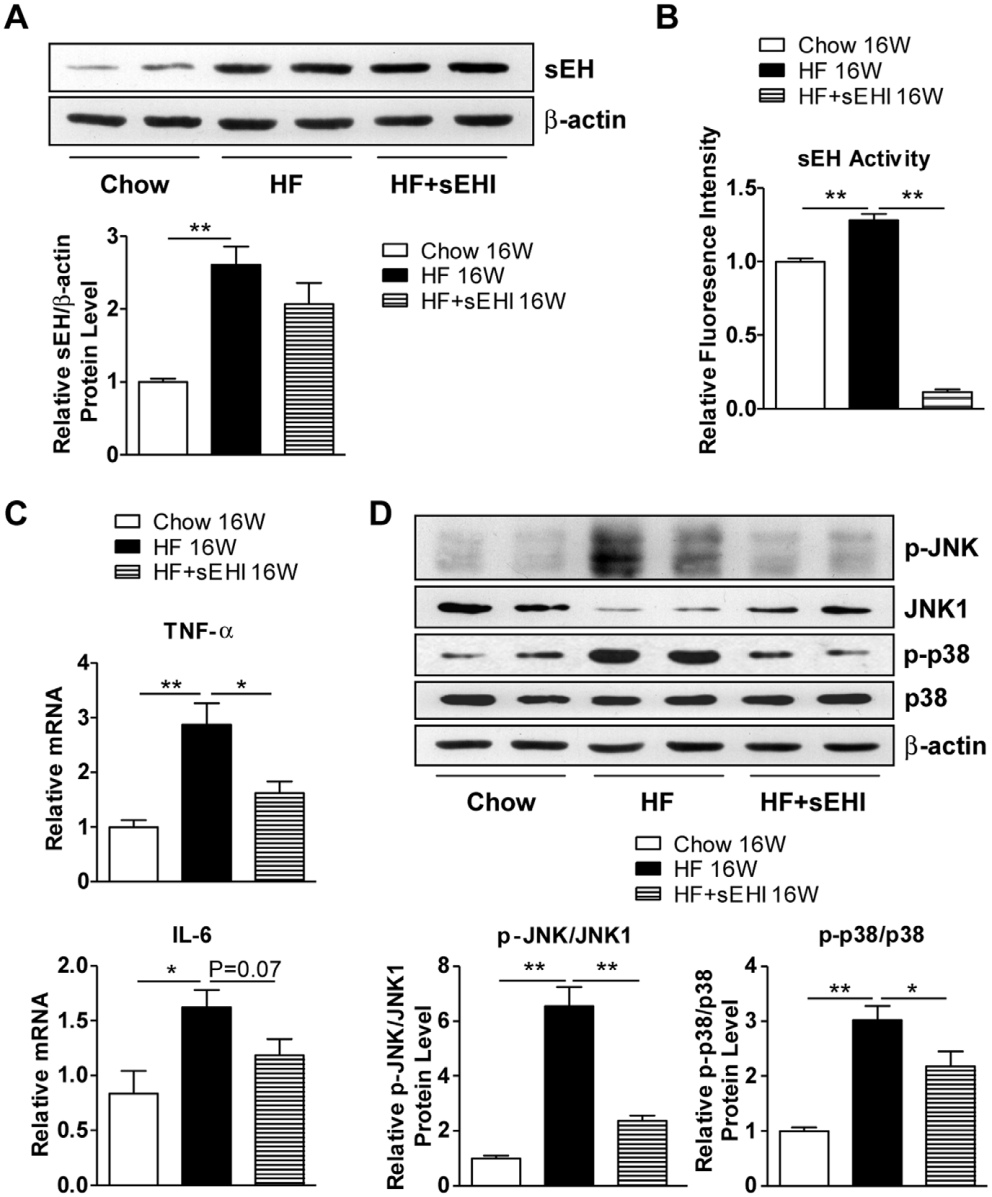
sEH inhibition decreased 16-week HF-diet–induced activation of inflammatory pathways in the liver. Male C57BL/6 mice were fed a regular chow or HF diet for 16 weeks with or without sEHI t-AUCB in drinking water starting 3 days before diet. (A) Western blot analysis of protein levels of sEH and β-actin as a normalization control in liver. (B) sEH activity in liver. (C) Quantitative RT-PCR analysis of mRNA levels of TNF-α and IL-6 in liver. (D) Western blot analysis of protein levels of phosphorylated Jun N-terminal kinase (p-JNK), JNK1, p-p38, p38 and β-actin and relative protein content compared to that of JNK1 or p38. Data are mean ± SEM. (* P<0.05, ** P<0.01).

**Figure 6:**
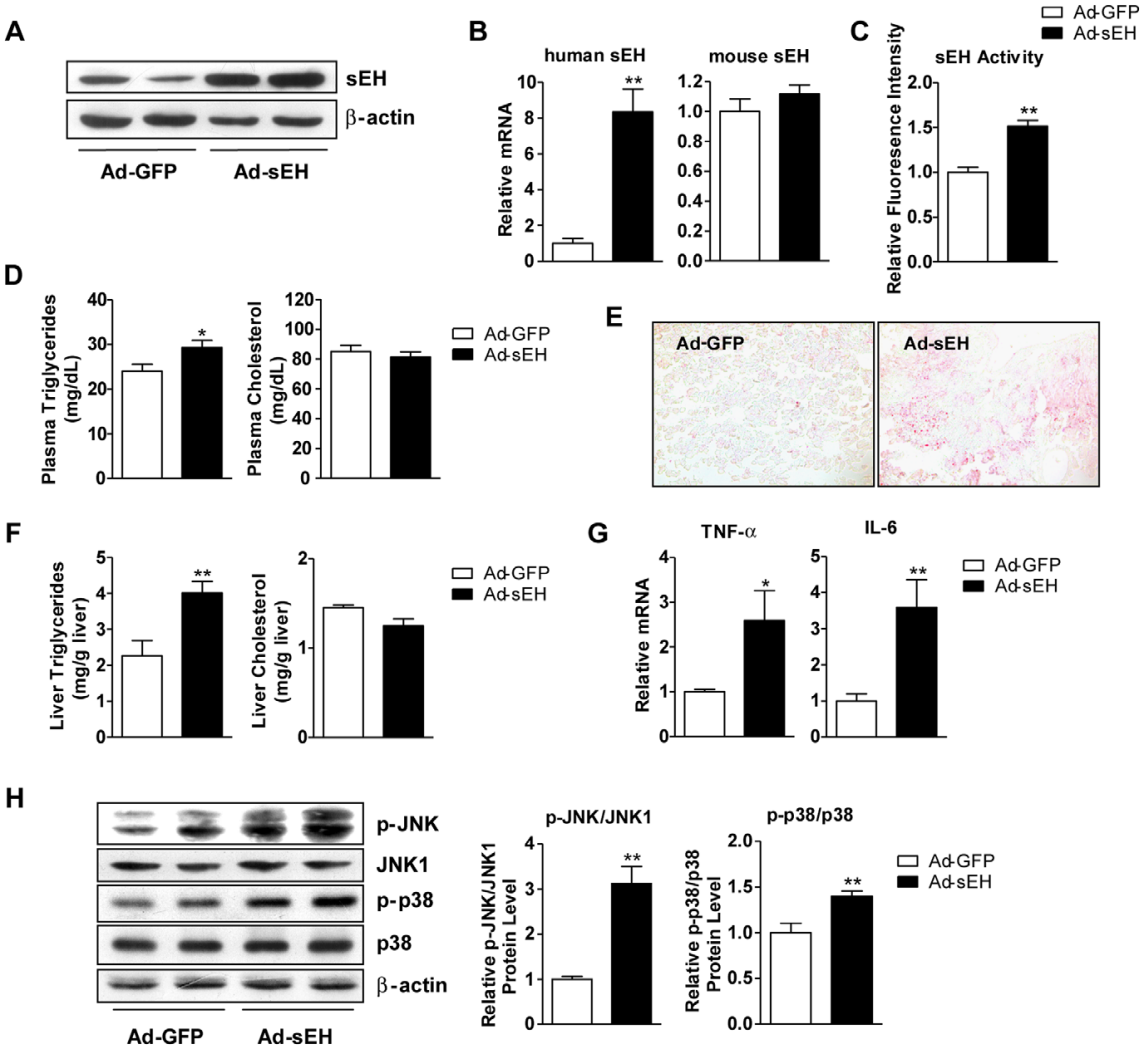
Hepatic sEH overexpression increased hepatic triglycerides accumulation. Male C57BL/6 mice were intravenously infected with 1×10^9^ PFU adenovirus (Ad)-sEH (n=7) or Ad-GFP (n=6) for 7 days. (A) Western blot analysis of sEH and β-actin in liver. (B) qRT-PCR analysis of mRNA levels of human sEH and mouse sEH. (C) sEH activity in liver. (D) Plasma levels of cholesterol and triglycerides. (E) Oil-red O staining in liver. (F) Levels of triglycerides and cholesterol in liver. (G) qRT-PCR analysis of mRNA levels of TNF-α and IL-6 in liver. (H) Western blot analysis of protein levels of p-JNK, JNK1, p38, p–p38 and β-actin and relative protein content compared to that of JNK1 or p38. Data are mean ± SEM. (* P<0.05, ** P<0.01).

We investigated the role of sEH in the pathogenesis of hepatic steatosis and found that as compared with an 8-week HF diet, a 16-week HF diet produced 2.6-fold increased sEH protein level in mouse liver (Fig. 5A) relative to the controls, with reinforced epoxide hydrolase activity of sEH (Fig. 5B). Furthermore, the long-term HF-diet activation of inflammatory pathways in the liver, including increased phosphorylation of JNK and p38 and mRNA levels of TNF-α and IL-6, was markedly reduced with sEH inhibition (Fig. 5C and D). To investigate the direct effect of sEH in the liver of mice, C57BL/6 mice were intravenously injected with recombinant adenoviruses encoding human sEH (Ad-sEH) or green fluorescence protein (Ad-GFP). At 7 days post-injection, sEH mRNA and protein levels and activity were higher in the liver of mice receiving Ad-sEH than the control Ad-GFP (Fig. 6A–C). The overexpression of sEH alone was sufficient for elevating the plasma and liver levels of triglycerides (Fig. 6D–F). Moreover, sEH overexpression increased the phosphorylation of JNK and p38 and the mRNA levels of TNF-α and IL-6 in the liver (Fig. 6G and H). Thus, the secondary upregulation of hepatic sEH with a long-term HF diet may play an important role in the progression of fatty liver and hepatic inflammation, from simple hepatic steatosis to steatohepatitis.

### sEHI Attenuated HF-diet–induced Macrophage Infiltration into Adipose Tissue

**Figure 7:**
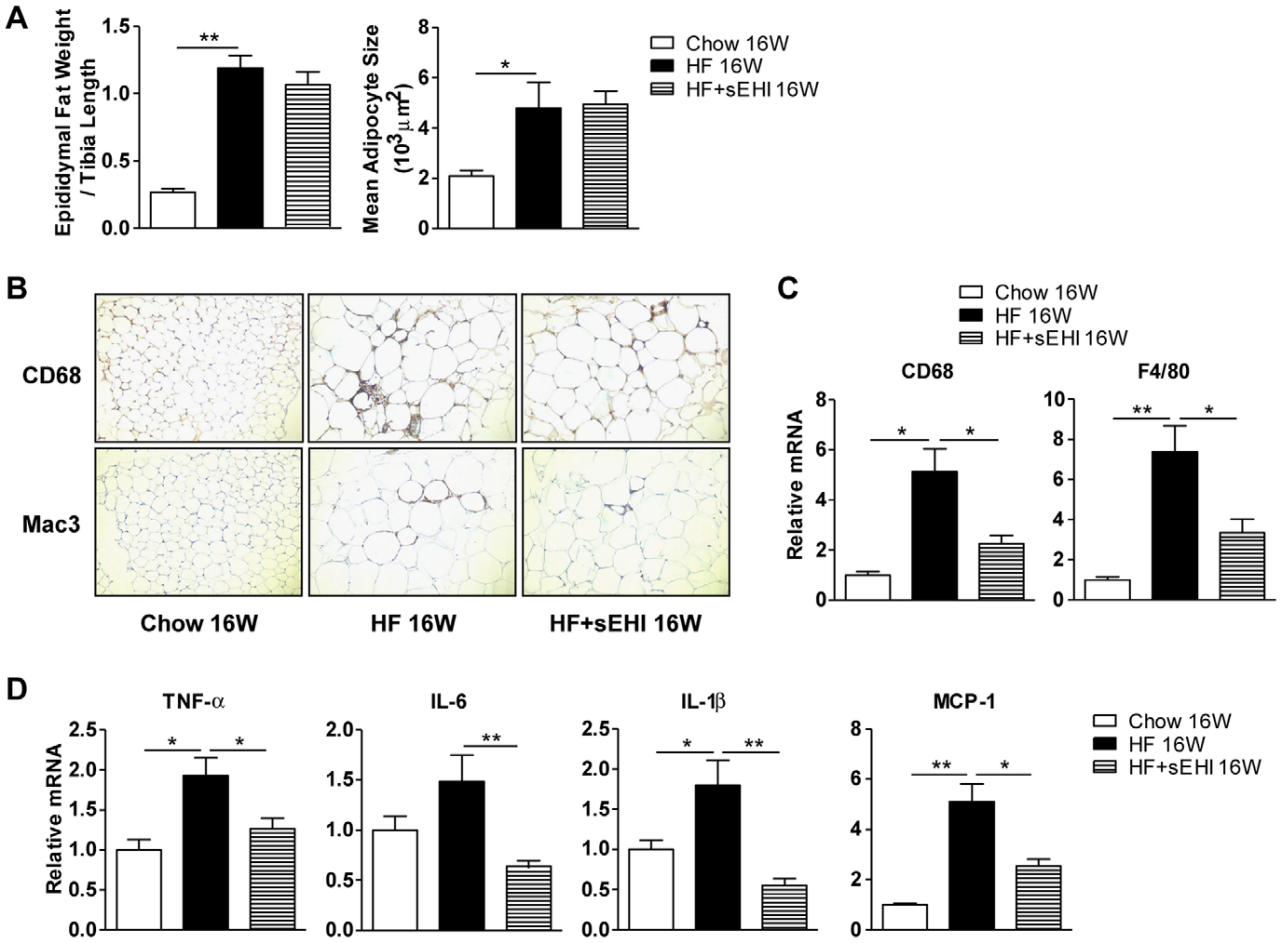
sEH inhibition attenuated 16-week HF-diet–induced macrophage infiltration in adipose tissue. (A) Ratio of epididymal fat weight compared to tibia length, and mean size of adiopocytes in epididymal fat of 16-week HF-diet–fed mice with or without full-term sEHI. (B) Immnohistochemical staining for macrophage markers CD68 and Mac3 in epididymal fat. (C) mRNA levels of CD68 and F4/80 in epididymal adipose tissue. (D) mRNA levels of TNF-α, IL-6, IL-1β and MCP-1 in epididymal adipose tissue. Data are mean ± SEM. (* P<0.05, ** P<0.01).

Given that sEH inhibition decreased macrophage infiltration and the expression of pro-inflammatory cytokines in adipose tissue with a short-term HF diet in mice, we investigated white adipose tissue of mice fed a 16-week HF diet. The size of adipocytes and weight of adipose tissue were increased with the diet but were not affected by sEH inhibition (Fig. 7A). However, sEH inhibition attenuated HF-diet–induced macrophage infiltration in epididymal fat tissue, as seen by staining for the macrophage markers CD68 and Mac3 (Fig. 7B), and increased mRNA levels of CD68 and F4/80 in adipose tissue (Fig. 7C). Consequently, the HF-diet–elevated mRNA expression of TNF-α, IL-6, IL-1β and MCP-1 was reduced with sEH inhibition (Fig. 7D), which was closely linked with their circulating levels.

## Discussion

sEH is broadly distributed in mammal tissues, with well-defined epoxide fatty acids, especially EETs, as excellent substrates. An optimal level of EETs has benefits such as dilating coronary arteries, suppressing adhesion molecule expression [24] and protecting against insulin resistance [25]. We and others reported that sEH inhibition relieved angiotensin II (AngII)-induced hypertension [26] and cardiac hypertrophy [27] and protected against apoptosis of pancreatic islet cells with streptozotocin-induced diabetes [28]. sEH inhibition decreased the formation of aortic atherosclerotic lesions with decreased expression of pro-inflammatory adhesion molecules in aortas of AngII-infused apolipoprotein E-null mice fed an atherogenic diet [24]. Given that the protein level of sEH was increased in aortas of AngII-infused Wistar-Kyoto rats and EETs had anti-inflammatory effects in vascular endothelial cells by inhibiting NF-κB and AP-1 expression [13], [29], the reduction in lesions was assumed to be associated with increased levels of EETs and the anti-inflammatory effect of sEH inhibition. Inflammation of local adipose tissue and inflammatory lipid mediators including EETs may play important roles in regulating adipocyte function and lipid metabolism [30]. sEH expression in adipose tissue did not differ between HF-diet–induced obese mice and controls, but total adipose sEH activity was increased, with a large increase in sEH expression during maturation of adipocytes [19]. A recent study reported that chronic oral treatment with t-AUCB could attenuate the signs of metabolic syndrome, including mild steatosis, in a rat model of metabolic syndrome [21], which supports our findings in mice with both pharmacological and genetic approaches. As well, we found that sEH overexpression in liver increased the inflammatory response, the expression of SREBP1 and its targets (data not shown), and the level of triglycerides in liver, as well as plasma level of triglycerides. Thus, in addition to systemic inflammation, increased sEH expression and activity may contribute to lipid accumulation in the liver. sEH inhibition may be important for controlling the pro-inflammatory response and symptoms of metabolic syndrome, including hepatic steatosis.

The reasons for HF-diet–induced hepatic steatosis are not clear. Following chronic excessive macronutrient intake, many metabolic changes, including body weight gain, excess visceral adipose deposition, insulin intolerance and system inflammation, may contribute to lipogenesis in liver. TNF-α and lipopolysaccharide treatment could accelerate hepatic fat accumulation in mice by upregulating the expression of SREBP1 and FAS [31]. Lee et**al. recently reported that inhibition of inflammation could not ameliorate short-term (3-day) HF-diet–induced lipid accumulation in the liver, but blocking macrophage infiltration had positive effects on protecting against long-term HF-diet–induced damage [32], which suggests the critical role of inflammation in chronic fatty liver disease. In our study, hepatic overexpression of sEH elevated the mRNA levels of TNF-α and IL-6 (Fig. 6G), which might contribute to the triglycerides accumulation in liver (Fig. 6F). HF diet for 16 weeks increased the hepatic expression of sEH protein, but the mechanism is unclear. To determine whether inflammation is responsible, we treated primary cultured mouse hepatocytes with the proinflammatory cytokines TNF-α or IL-6 for 24 hr and observed elevated protein level of sEH (data not shown). Hence, the increased sEH expression might be due to elevated proinflammatory response to the HF diet. However, with an HF diet for 8 weeks, the liver expression of sEH and enzymes involved in fatty acid synthesis were not changed, and the mRNA level of FAS was lower. The paradoxical lipid-related gene profile in liver with an HF diet could be due to feedback suppression. Nevertheless, sEH inhibition did not alter the hepatic lipid-related gene profile, which suggested an indirect role of sEH inhibition in regulating hepatic lipid metabolism in obesity, and inhibition of inflammation in peripheral tissues could not be excluded.

Obesity involves chronic inflammation, with increased macrophage infiltration in adipose tissue and secretion of various cytokines [33], [34]. It is associated with many metabolic disorders, including steatosis and steatohepatitis. Low-grade circulating and adipose tissue levels of TNF-α and IL-6 were found positively correlated in obese women, which suggested circulating proinflammatory cytokines originating from adipose tissue in obese individuals [35]. Cytokines could also be secreted by infiltrated macrophages in adipose tissue as well as adipocytes. We found that sEH inhibition attenuated HF-diet–induced macrophage infiltration and elevated mRNA levels of TNF-α, IL-6, IL-1β and MCP1 in adipose tissue (Fig. 7), as well as reduced the circulating levels of these cytokines. The size of adipocytes and weight of adipose tissue were not regulated by sEH inhibition, so the decreased proinflammatory cytokine secretion might mainly result from reduced macrophage infiltration in adipose tissue.

Moreover, sEH inhibition or overexpression might result in a shift of arachidonic acid metabolic pathways: with one pathway inhibited, other pathways may be activated. Using a metabolomic approach, we previously demonstrated that chronic administration of a selective cyclooxygenase 2 inhibitor resulted in >120-fold increase in blood level of 20-hydroxyeicosatetraenoic acid, a cytochrome P450 (CYP450) metabolite of arachidonic acid [36]. In addition, omega-3 polyunsaturated fatty acids are substrates of CYP450 epoxgenases [37], and the epoxy products of DHA and EPA could be hydrolyzed by sEH. The epoxy metabolite of EPA, 17, 18-epoxyeicosatetraenoic acid, showed anti-inflammatory effects by decreasing the contractile reactivity and Ca^2+^ sensitivity of TNF-α–pretreated human bronchi [38]. Overexpression of CYP2J3 increased the generation of EETs and reduced insulin resistance in both db/db mice and fructose-treated diabetic rats [39]. Thus, the change in levels of CYP450 metabolites of omega-3 fatty acids, along with EETs, mediated by hepatic sEH overexpression might contribute to increased inflammatory reaction. Metabolite alteration by sEH inhibition mediating the inflammation and fatty liver should not be excluded.

**Figure 8:**
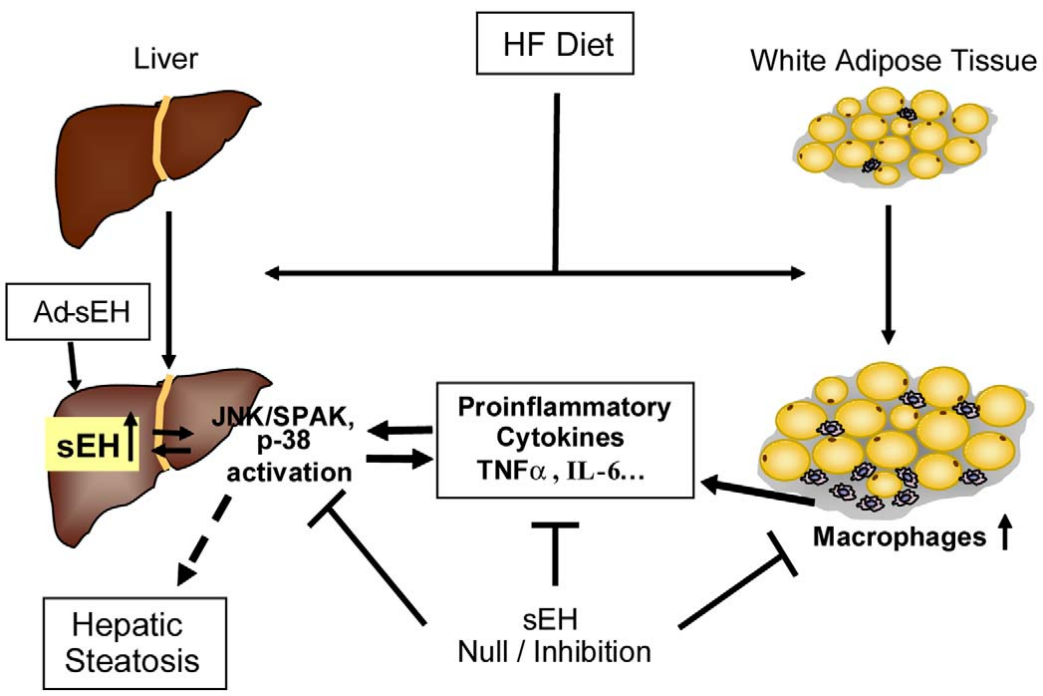
Proposed model for the regulation of hepatic steatosis by sEH in mice. Long-term HF diet increases sEH protein level in the liver and leads to hepatic lipid accumulation and obesity with increased macrophage infiltration in white adipose tissue. sEH inhibition or deficiency reduces HF-diet–induced hepatic steatosis by inhibiting systemic inflammation, especially in adipose tissue and liver (long term) as one possible mechanism. sEH overexpression in the liver with adenovirus injection increased inflammation and hepatic lipid accumulation.

We propose a model of crosstalk between liver and adipose tissue for lipid metabolism in the liver regulated by an HF diet and sEH inhibition (Fig. 8). In the early stage of HF-diet–induced obesity, inflammation is increased because of inflammatory cell infiltration, and circulating cytokines such as TNF-α and IL-6 are elevated; the latter further activates inflammatory signaling in the liver, which in turn, promotes hepatic lipid accumulation. A long-term HF diet also upregulates hepatic sEH expression, which may accelerate HF-diet–induced hepatic damage. sEH inhibition or sEH knockout improved the inflammatory status in adipose tissue, thus reduced circulating proinflammatory cytokines in the early stage of HF diet feeding. Inhibiting the upregulated hepatic expression of sEH may ameliorate the steatosis induced by a chronic HF diet treatment. Thus, HF-diet–induced obesity increased the systemic proinflammatory status, which upregulated hepatic sEH expression and activated inflammatory signaling in the liver and in turn, promoted hepatic lipid accumulation. By attenuating inflammation in both fat tissue and liver, sEH inhibition may provide a therapeutic strategy in the treatment of NAFLD.

## Materials and Methods

### Ethics Statement and Animal Experimental Procedure

The investigation conformed to the Guide for the Care and Use of Laboratory Animals by the US National Institutes of Health (NIH Publication No. 85–23, revised 1996). The animal experimental protocol was approved by the Institutional Animal Care and Use Committee of the Peking University Health Science Center (LA2011–003). Mice with targeted disruption of *Ephx2* gene (sEH null) [22] were back-crossed onto a C57BL/6 genetic background for more than ten generations as previously described [40]. Eight-week-old male sEH-null mice and their WT littermates and C57BL/6 mice were maintained under controlled temperature and a 12-h light/dark cycle, with free access to water and standard laboratory chow. Mice were divided into groups for different diets and treatment as indicated. Each group of sEH-null and WT mice (n≥6 each) or C57BL/6 mice (n≥8) were fed a chow diet (10 kcal% fat, D12450B) or a protein-matched HF diet (60 kcal% fat, D12492, both Research Diets, New Brunswick, NJ, USA). For sEH inhibitor (sEHI) treatment, 2 mg t-AUCB (synthesized in Dr. Bruce D Hammock’s laboratory) was added to 0.5 ml PEG400 and ultrasonicated until completely dissolved, and then given to mice (20 mg/L in 100 ml tap drinking water, starting time as indicated). At the last week of diets, mice were intraperitoneally injected with glucose (1 g glucose/kg body weight) or insulin (1 U/kg body weight) after an overnight or a 6-hr fast, and blood glucose was determined by use of a portable glucometer (ACCU-CHEK II, Roche Diagnostics, Mannheim, Germany). At the end of treatment, all animals were anesthetized and killed after a 6-hr fast. Plasma was harvested for measuring plasma levels of lipids, insulin and cytokines. Plasma insulin was measured by use of an ELISA kit (Millipore, Billerica, MA, USA) or an ultrasensitive I^125^-linked immunosorbent assay kit (Fu Rui Inc., Beijing). Tissues were collected and immediately snap-frozen in liquid nitrogen, and stored at -80^°^C.

### Adenovirus Purification and Infection *In vivo*

Recombinant adenoviruses encoding green fluorescence protein (Ad-GFP) and human sEH (Ad-sEH) were constructed and amplified as described [27]. Virus particles were purified by cesium chloride gradient and concentrated by use of Sephadex-G-25M columns (GE Healthcare, Piscataway, NJ, USA). Titers of virus (plaque-forming units [PFUs]) were determined in HEK293 cells. For adenoviral infection, 12-week-old C57BL/6 mice were injected with Ad-GFP or Ad-sEH (n≥6) in the tail vein (1×10^9^ PFUs diluted in 0.1 ml saline), then fed a regular diet for the next 7 days before being killed.

### Measurement of Lipids in Plasma and Liver

The plasma levels of triglycerides and cholesterol were measured by use of an automated clinical chemistry analyzer kit (Biosino Biotech, Beijing). For quantification of hepatic triglycerides and cholesterol levels in liver, tissues were homogenized, and total lipids were extracted by use of a 2∶1 mixture of chloroform: methanol. Dried lipid residues were dissolved in phosphate buffered saline containing 5% Triton X−100, and levels of triglycerides and cholesterol were determined with use of the same kit used for plasma analysis.

### Immunohistochemistry and Oil-red O Staining

Liver and epididymal adipose tissue was fixed, dehydrated, and embedded, and then blocks underwent sectioning. Frozen liver sections were stained with Oil-red O. Adipose tissue sections were incubated with the mouse primary antibody against CD68 (1∶200, Abcam, Cambridge, UK) or rat antibody against Mac3 (1∶100; Santa Cruz Biotechnology, Santa Cruz, CA, USA) overnight at 4°C, then anti-mouse or -rat horseradish peroxidase-conjugated secondary antibody (Zhong-shan Golden Bridge, Beijing) for 1 hr at 37°C. The nuclei were counterstained with haematoxylin. Slides were photographed by use of an Eclipse TE2000 inverted microscope system (Nikon Instruments, Melville, NY, USA) at ×200 magnification.

### Western Blot Analysis

Equal amounts of liver protein were separated on 10% SDS-PAGE and transferred onto PVDF membrane (Millipore), which was incubated with primary antibodies against sEH (Cayman Chemical, Ann Arbor, MI, USA), phospho-p38 (Bioworld, Louis Park, MN, USA), p38, phospho–stress-activated protein kinase (SAPK)/Jun N-terminal kinase (JNK) (Cell Signaling, Danvers, MA, USA), total SAPK/JNK1 or β-actin (Santa Cruz Biotechnology), then horseradish peroxidase-conjugated anti-rabbit or anti-mouse IgG antibody (Cell Signaling), and underwent chemiluminescence detection (Engreen Biosystem, Beijing).

### Real-time PCR Analysis

Total RNA in liver and epididymal adipose tissue was isolated by the Trizol reagent method (Invitrogen, Carlsbad, CA, USA) and reverse transcribed by use of the First Strand cDNA Synthesis kit (Thermo Scientific, Rockford, IL, USA). The amplification reactions were in a volume of 20 µl consisting of synthesized cDNA, primers and EasyTaq PCR Mix (Transgen Biotech, Beijing). Eva Green was used to monitor amplification of DNA by the MX3000P qPCR detection system (Stratagene, Santa Clara, CA, USA). Fold change in mRNA concentration was calculated by the comparative C_T_ method. Gene expression was normalized to β-actin levels. The sequences of primers are in Table S1.

### sEH Activity Assay

Liver tissues were homogenized in deionized water with 1 mM PMSF, and cytosolic supernatants were obtained by centrifugation. In total, 100 µl of samples were incubated with or without 20 µM t-AUCB at 30°C for 10 min in 96-well black assay plates, and 80 µM epoxy fluor 7 in 100 µl reaction buffer (50 mM Tris-Hcl buffer containing 0.2 mg/ml bovine serum albumin, pH 7.0) was added to each well. After incubation at 30°C for another 30 min, fluorescence was determined by use of a Spectra Fluor Plus Xuorescent plate reader (Tecan Systems, San Jose, CA, USA) with excitation wavelength 330 nm (bandwidth, 20 nm), emission wavelength 465 nm (bandwidth, 20 nm), and temperature 30°C [41]. sEH activity was the corrected sample fluorescence intensity calculated by sample fluorescence values for the sEH inhibitor subtracted from those of the non-sEH inhibitor.

### Cytokine Measurement

The plasma levels of TNF-α, IL-6, MCP-1 and IFN-γ were measured by Cytometric Bead Array with use of a mouse inflammation kit (BD, San Jose, CA, USA).

### Statistical Analyses

The significance of variability was evaluated by unpaired two-tailed Student’s *t* test. P<0.05 was considered statistically significant.

## Supporting Information

Figure S1 **4-week sEH inhibition or sEH knockout did not improve HF-diet–induced insulin resistance.** C57BL/6 mice were fed an 8-week regular-chow or HF diet with or without sEH inhibitor (sEHI) t-AUCB. (A) Intravenous glucose tolerance test. (B) Plasma level of insulin. (C) Insulin resistance index (IRI) calculated by plasma insulin level and fasting blood glucose. sEH wild-type (WT) or sEH-null mice were fed an 8-week regular-chow or HF diet. (D) Intravenous glucose tolerance test. (E) Plasma level of insulin. (F) Insulin resistance index (IRI) calculated by plasma insulin level and fasting blood glucose level. Data are mean ± SEM. (* P<0.05).
(TIF)

Figure S2 **Expression of genes in the liver of mice.** C57BL/6 mice were fed an 8-week regular-chow or HF diet with or without sEH inhibitor (sEHI) t-AUCB. qRT-PCR analysis of mRNA levels in liver of genes involved in (A) fatty acid synthesis, LXR-α, SREBP1, ChREBP, FAS and ACC; and (B) fatty acid β-oxidation, PPAR-α, CPT1A and ACO1; and (C) inflammation, TNF-α and IL-6. Data are mean ± SEM. (* P<0.05, ** P<0.01).
(TIF)

Figure S3 **sEH inhibition did not improve 16-week HF-diet–induced insulin resistance.** Mice fasted for 6 hr from 9∶00 am at the end of the week 15, and then were tested for (A) intravenous glucose tolerance and (B) insulin glucose tolerance. (C) ELISA of plasma level of insulin. (D) Insulin resistance index (IRI) was calculated with fasting glucose level and plasma insulin level. Data are mean ± SEM. (* P<0.05, ** P<0.01).
(TIF)

Table S1 Primers used in this study.
(PDF)
