# Supplementary figures and images for: Inhibition of Soluble Epoxide Hydrolase Attenuates High-Fat-Diet–Induced Hepatic Steatosis by Reduced Systemic Inflammatory Status in Mice
Source: PLoS One. 2012 Jun 14;7(6):e39165. doi: 10.1371/journal.pone.0039165 (PMC3375303; doi:10.1371/journal.pone.0039165)

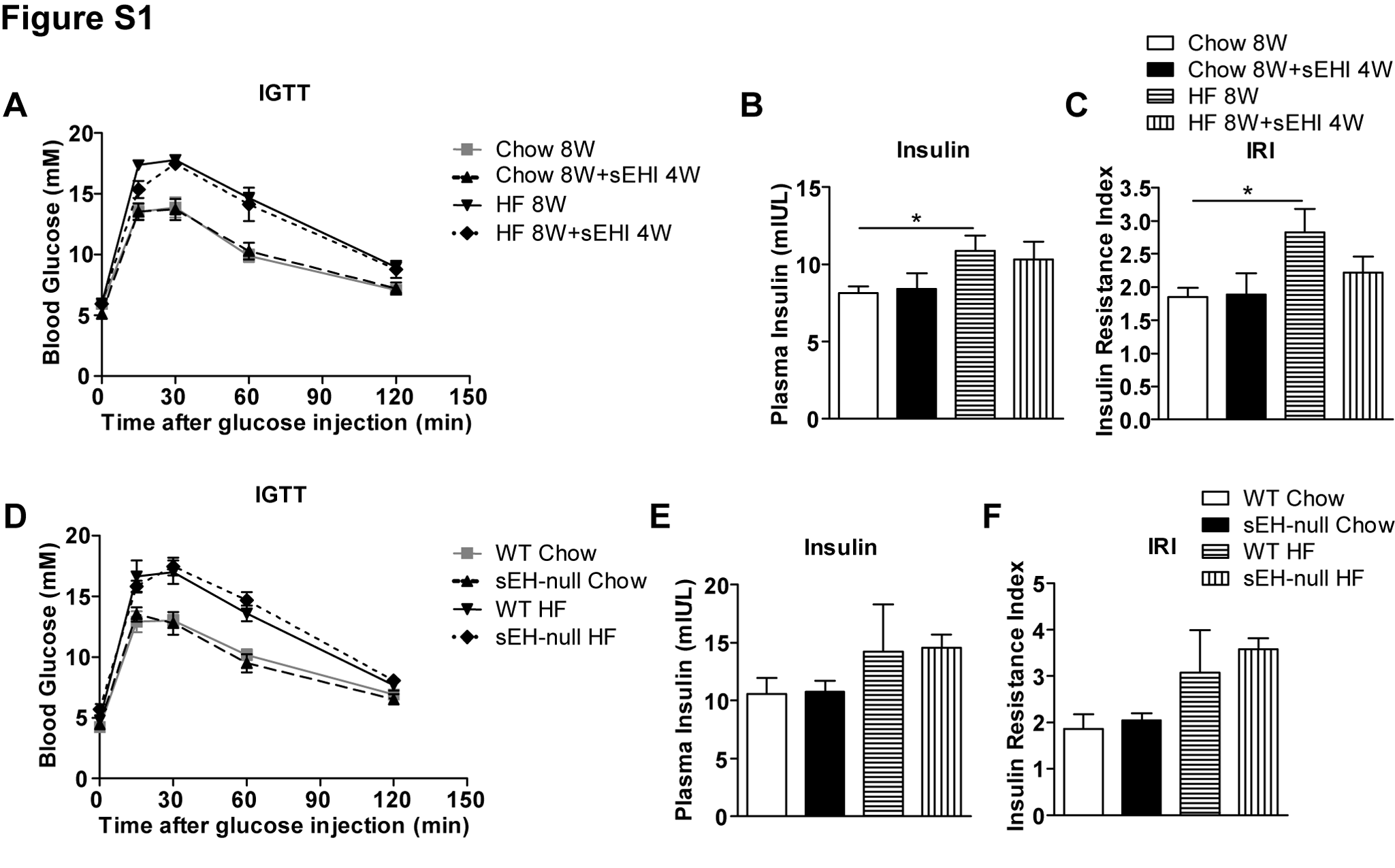

Supplement: Figure S1 — 4-week sEH inhibition or sEH knockout did not improve HF-diet–induced insulin resistance. C57BL/6 mice were fed an 8-week regular-chow or HF diet with or without sEH inhibitor (sEHI) t-AUCB. (A) Intravenous glucose tolerance test. (B) Plasma level of insulin. (C) Insulin resistance index (IRI) calculated by plasma insulin level and fasting blood glucose. sEH wild-type (WT) or sEH-null mice were fed an 8-week regular-chow or HF diet. (D) Intravenous glucose tolerance test. (E) Plasma level of insulin. (F) Insulin resistance index (IRI) calculated by plasma insulin level and fasting blood glucose level. Data are mean ± SEM. (* P<0.05). (TIF) [file pone.0039165.s001.tif]

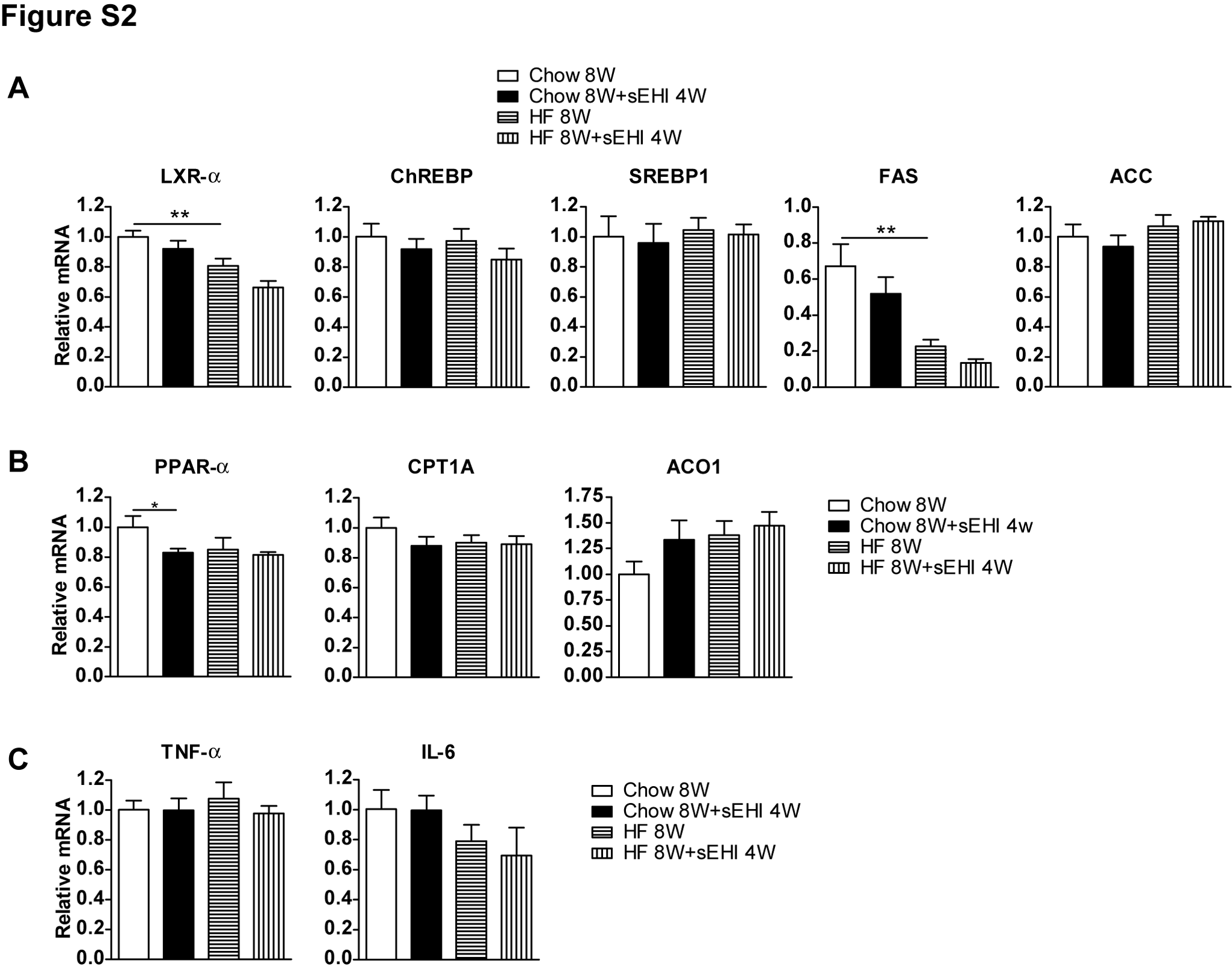

Supplement: Figure S2 — Expression of genes in the liver of mice. C57BL/6 mice were fed an 8-week regular-chow or HF diet with or without sEH inhibitor (sEHI) t-AUCB. qRT-PCR analysis of mRNA levels in liver of genes involved in (A) fatty acid synthesis, LXR-α, SREBP1, ChREBP, FAS and ACC; and (B) fatty acid β-oxidation, PPAR-α, CPT1A and ACO1; and (C) inflammation, TNF-α and IL-6. Data are mean ± SEM. (* P<0.05, ** P<0.01). (TIF) [file pone.0039165.s002.tif]

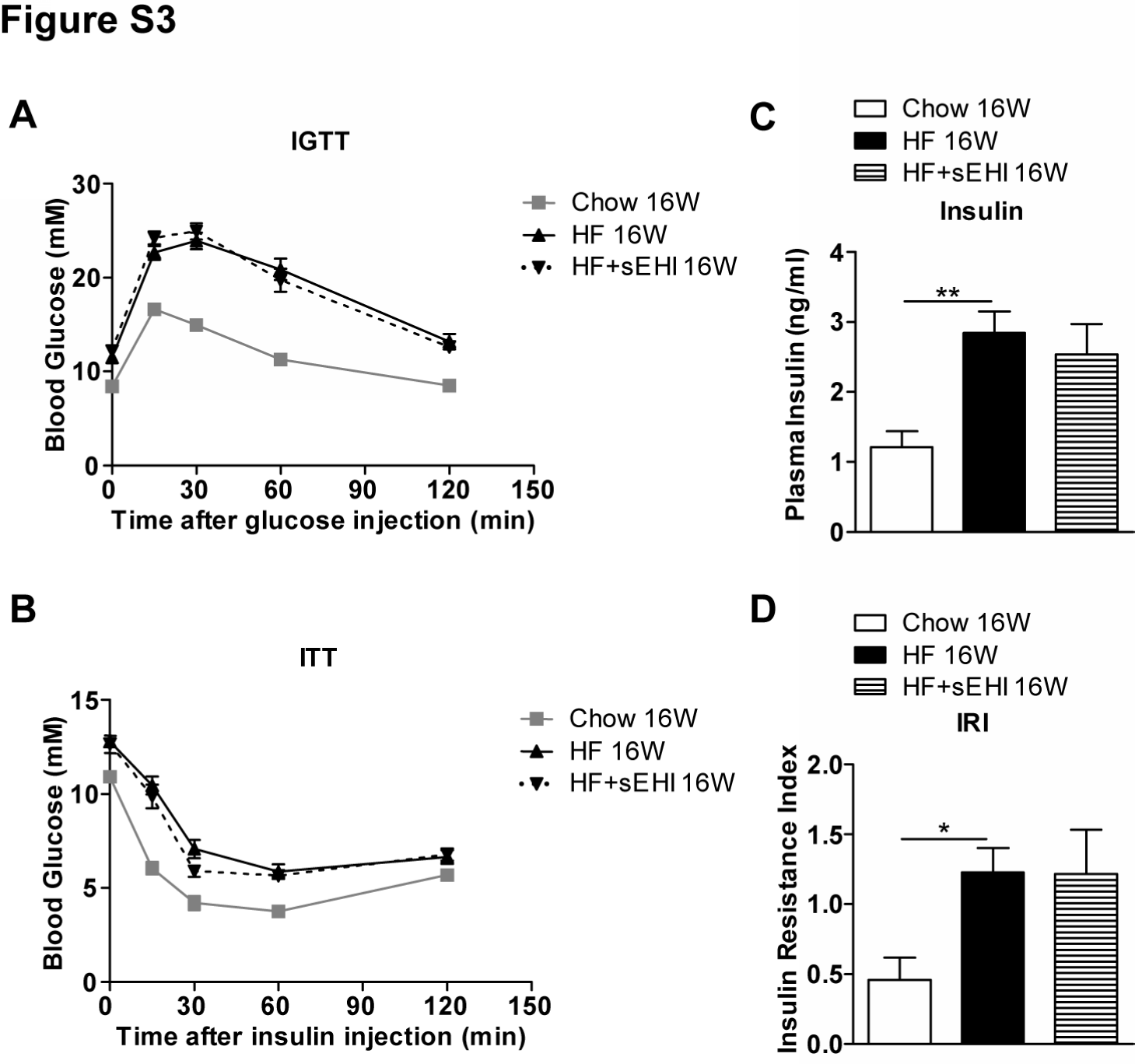

Supplement: Figure S3 — sEH inhibition did not improve 16-week HF-diet–induced insulin resistance. Mice fasted for 6 hr from 9∶00 am at the end of the week 15, and then were tested for (A) intravenous glucose tolerance and (B) insulin glucose tolerance. (C) ELISA of plasma level of insulin. (D) Insulin resistance index (IRI) was calculated with fasting glucose level and plasma insulin level. Data are mean ± SEM. (* P<0.05, ** P<0.01). (TIF) [file pone.0039165.s003.tif]
