# Supplementary material for: Inhibition of Soluble Epoxide Hydrolase Attenuates High-Fat-Diet–Induced Hepatic Steatosis by Reduced Systemic Inflammatory Status in Mice
Source: PLoS One. 2012 Jun 14;7(6):e39165. doi: 10.1371/journal.pone.0039165 (PMC3375303; doi:10.1371/journal.pone.0039165)
Supplement: Table S1 — Primers used in this study. (PDF) [file pone.0039165.s004.pdf]

Table S1

Primers for quantitative RT-PCR				
**Mouse & Human**	**NM**	**nt**	**Forward (5' to 3')**	**Reverse (5' to 3')**
β-*actin* (β-actin)	NM_001101	291	ATCTGGCACCACACCTTC	AGCCAGGTCCAGACGCA
**Human**	**NM**	**nt**	**Forward (5' to 3')**	**Reverse (5' to 3')**
EPHX2 (sEH)	NM_001256484	219	TGTAAATAGCCCAGAAGAGC	TGAGGAACGAGCACGAA
**Mouse**	**NM**	**nt**	**Forward (5' to 3')**	**Reverse (5' to 3')**
Ephx2 (sEH)	NM_007940	136	GGTGACATTCCTGGATAAG	AAACGGCGTGTTCAAACT
Lxra (LXR-α)	NM_013839	161	TGCCATCAGCATCTTCTCTG	GGCTCACCAGCTTCATTAGC
Srebf1 (SREBP1)	NM_011480	279	ACTTCTGGAGACATCGCAAAC	GGTAGACAACAGCCGCATC
Mlxipl (ChREBP)	NM_021455	189	TTACTGGAAGCGGCGCATCG	CCAAGCAGCACAGGCACCAC
Fasn (FAS)	NM_007988	158	TGGGTTCTAGCCAGCAGAGT	ACCACCAGAGACCGTTATGC
Acaca (ACC)	NM_133360	206	TGG TCG TGACTGCTCTGTGC	GTAGCC GAGGGT TCAGTTCC
Ppara (PPAR-α)	NM_011144	55	CGTACGGCAATGGCTTTATC	AACGGCTTCCTCAGGTTCTT
Cpt1a (CPT1A)	NM_013495	65	TGAGTGGCGTCCTCTTTGG	CAGCGAGTAGCGCATAGTCA
Acox1 (ACO1)	NM_015729	102	TCGTGGGAAGTGCAGCTC AG	GCT CTG GCT CGC TTC TCT TG
Emr1 (F4/80)	NM_010130	165	CTTTGGCTATGGGCTTCCAGTC	GCAAGGAGGACAGAGTTTATCGTG
Cd68 (CD68)	NM_009853	190	CCCAAGGAACAGAGGAAG	GTGGCAGGGTTATGAGTG
Tnfa (TNF-α)	NM_013693	79	CCAGACCCTCACACTCAGATC	CACTTGGTGGTTTGCTACGAC
Il6 (IL-6)	NM_031168	129	ACAACCACGGCCTTCCCTACTT	CACGATTTCCCAGAGAACATGTG
Ccl2 (MCP-1)	NM_011333	249	AGGTCCCTGTCATGCTTCTG	TCTGGACCCATTCCTTCTTG
Il1b (IL-1β)	NM_008361	230	GCCCATCCTCTGTGACTCAT	AGGCCACAGGTATTTTGTCG
Ifng (IFN-γ)	NM_008337	92	TCAAGTGGCATAGATGTGGAAGAA	TGGCTCTGCAGGATTTTCATG
